# Anticipatory care planning intervention for older adults at risk of functional decline: study protocol for a primary care cluster feasibility randomised trial

**DOI:** 10.1186/s13063-020-4100-2

**Published:** 2020-02-11

**Authors:** Kevin Brazil, David Scott, Emma Wallace, Mike Clarke, Tom Fahey, Patrick Gillespie, Peter O’Halloran, Christopher Cardwell, Gillian Carter, Kieran McGlade, Frank Doyle

**Affiliations:** 1 School of Nursing and Midwifery, Queen’s University of Belfast, 97 Lisburn Road, Belfast, Northern Ireland BT9 7BL UK; 2 Royal College of Surgeons in Ireland, 123 St Stephen’s Green, Dublin, D02 YN77 Ireland; 3 Centre for Public Health, ICSB, Royal Victoria Hospital, Queen’s University Belfast, Belfast, Northern Ireland BT12 6BA UK; 4 Health Economics and Policy Analysis Centre, National University of Ireland, Galway, H91 TK33 Ireland; 5 Centre for Medical Education, School of Medicine, Dentistry and Biomedical Sciences, Queen’s University Belfast, 97 Lisburn Road, Belfast, BT9 7BL UK; 6 School of Psychology, Queen’s University Belfast, University Road, Belfast, Northern Ireland BT71NN UK

**Keywords:** Cluster randomised controlled trial, Anticipatory care planning, Primary care, Frailty, Functional decline

## Abstract

**Background:**

The treatment and management of long-term health conditions is the greatest challenge facing health systems around the world today. Innovative approaches to patient care in the community such as Anticipatory Care Planning (ACP), which seek to help with the provision of high-quality comprehensive care to older adults at risk of functional decline, require evaluation. This study will evaluate one approach that will include primary care as the setting for ACP.

**Methods/design:**

This study will help to determine the feasibility for a definitive randomised trial to evaluate the implementation and outcomes of an ACP intervention. The intervention will be delivered by specially trained registered nurses in a primary care setting with older adults identified as at risk of functional decline. The intervention will comprise: (a) information collection via patient assessment; (b) facilitated informed dialogue between the patient, family carer, general practitioner and other healthcare practitioners; and, (c) documentation of the agreed support plan and follow-up review dates. Through a structured consultation with patients and their family carers, the nurses will complete a mutually agreed personalised support plan.

**Discussion:**

This study will determine the feasibility for a full trial protocol to evaluate the implementation and outcomes of an (ACP) intervention in primary care to assist older adults aged 70 years of age or older and assessed as being at risk of functional decline. The study will be implemented in two jurisdictions on the island of Ireland which employ different health systems but which face similar health challenges. This study will allow us to examine important issues, such as the impact of two different healthcare systems on the health of older people and the influence of different legislative interpretations on undertaking cross jurisdictional research in Ireland.

**Protocol version:**

Version 1, 17 September 2019.

**Trial registration:**

Clinicaltrials.gov, ID: NCT03902743. Registered on 4 April 2019.

## Background

The delivery of high-quality, comprehensive care for older adults is becoming an increasing challenge for health systems around the world due to the ageing of society, increasing levels of multimorbidity with complex polypharmacy, shortages of healthcare providers, and rising healthcare costs [1–5]. Within the healthcare landscape primary care is increasingly seen as the optimal context to deliver care for people with complex care needs because it is accessible, efficient and can address inequalities related to socioeconomic deprivation. However, the current approach in primary care is seen as reactive and fragmented and does not fully meet the often complex needs of older adults [3, 6, 7]. A transition toward the provision of more proactive care in primary care has been proposed [3, 6, 7]. Proactive primary care requires a timely start to the identification and management of a patient’s long-term conditions or chronic illnesses. This process can be facilitated using Anticipatory Care Planning (ACP) to meet a patient’s wishes and needs, relieve symptoms, and prevent future symptoms and problems. A core aspect of anticipatory care is personalised care planning [8] which aims to ensure that an individual’s values and health concerns inform the way that their long-term conditions are managed. Instead of focussing on a standardised set of disease management processes determined by health professionals, this personalised approach encourages patients to select treatment goals and to work with clinicians to determine their specific needs for treatment and support. When considering the introduction of ACP in primary care there is a need to identify information about strategies for patient risk stratification, which service components or service innovations are beneficial and, which are redundant.

This study examines one approach to the provision of ACP in a primary care setting. Using a randomised design, it will determine the feasibility of undertaking a definitive trial to evaluate the implementation and outcomes of an ACP intervention in primary care. The intervention is designed to assist older adults identified as being at risk of functional decline by developing a personalised support plan. The outcomes from this feasibility cluster randomised controlled trial (cRCT) will allow us to determine the methodological and statistical considerations required to move forward with the definitive trial. Furthermore, we will be able to examine potential improvements in care for older persons at risk of functional decline such as: improved ability to assess care needs and respond appropriately; enhance decision-making among patients regarding their care; and, improve communication between patients, their family carers and healthcare providers on identified goals of care. In addition, this study should help us to determine if it is possible and appropriate to position the intervention within a primary care setting. It should also be noted that the study includes a cross-border approach, with primary care settings in the Republic of Ireland (RoI) and Northern Ireland (a region within the United Kingdom). This provides an opportunity to identify optimal system delivery supports for effective implementation in different healthcare systems and their potential impact on the delivery and outcomes of the ACP intervention.

## Methods/design

### Study overview

**Fig. 1:**
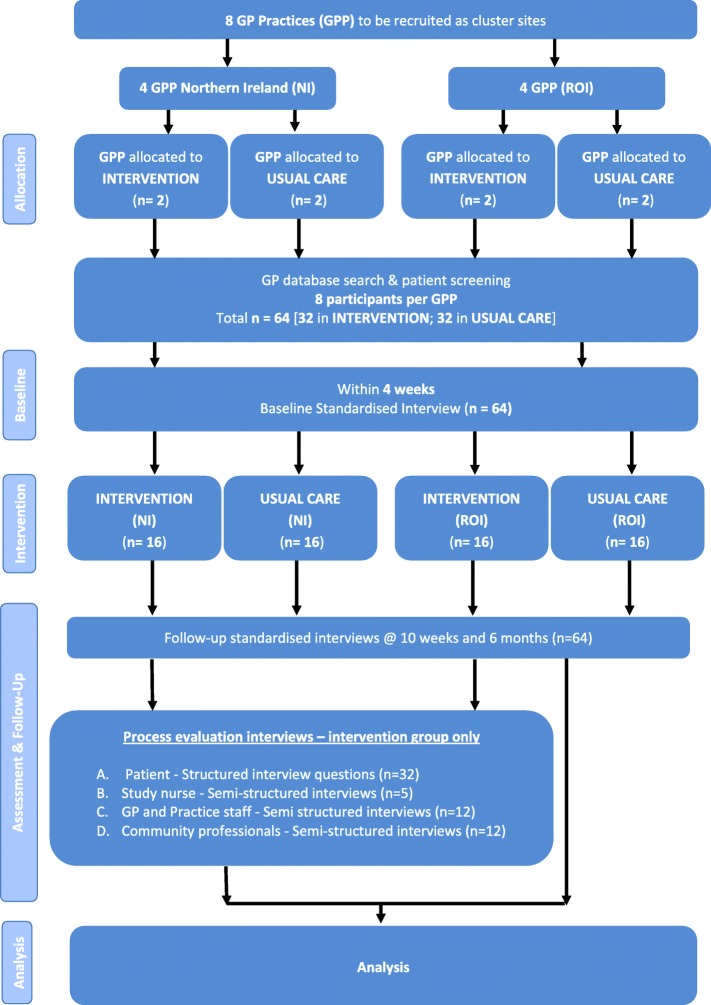
Study procedures

In order to examine the feasibility of this ACP intervention we will perform an open-label feasibility cRCT where eight general practitioner (GP) practices will be randomly assigned at a 1:1 ratio (four practices per group) to the intervention (ACP) or usual care alone. The randomisation will be stratified by jurisdiction (Northern Ireland and the RoI) and, in the RoI, by county (Cavan and Louth). GP practices will be randomly allocated to the intervention or usual care arms of the study before the screening of patients for signs of functional decline. An overview of the trial is presented in Fig. 1, the World Health Organisation (WHO) Registry Checklist is available (Additional file 1) and we follow the Standard Protocol Items: Recommendations for Interventional Trials (SPIRIT) Checklist (Additional file 2).

### Recruitment of study centres

Practices in Northern Ireland will be recruited via the Northern Ireland Clinical Research Network (Primary Care), an initiative which aims to facilitate the completion of clinical trials within the National Health Service (NHS). In the RoI, informal and formal networks, such as the national Health Research Board (HRB) Primary Care Trials Network, will assist in recruiting general practices located in the border counties of Louth and Cavan.

The allocation of general practices to intervention or control will not be revealed until the practice has formally entered the trial, thereby maintaining allocation concealment. A general practice will be ready for randomisation when it has completed the screening of its patient list to identify those who are eligible for the study. Each general practice will have a 50% probability of being allocated to the intervention or to the control group, with two intervention and two control practices being identified in each jurisdiction. The random allocation to intervention or control group will be undertaken when all four general practices in the relevant jurisdiction have completed screening and are ready for randomisation.

#### Randomisation

As a first step, the trial administrator will order the GP practices alphabetically and use this order to code the four practices in Northern Ireland and the four practices in the RoI. The trial administrator will inform the co-applicant methodologist when all four practices in Northern Ireland or the RoI are ready for randomisation and send him their codes, along with an indication of whether the practice is urban or rural. The methodologist, who will be blind to the identity of the practice, will then use the random number function in Microsoft Excel to assign a random number for each code. In each jurisdiction, the urban and the rural practice with the lowest number will be allocated to the intervention group and the other two practices will be allocated to the control group. The methodologist will send the allocations to the trial administrator, who, by linking the alphabetic code to the relevant practice, will know to which group each practice has been allocated. The trial administrator will inform the research nurse of the allocations and will then inform each practice of their allocation.

#### Setting

The project will be implemented across two healthcare systems: Northern Ireland and the RoI. Northern Ireland is a region within the United Kingdom that provides an integrated health and social services model of care, under the NHS, which is free to the user at the point of delivery. The RoI has a mixed public-private healthcare system with all persons resident in the country entitled to receive hospital care through the publicly funded healthcare system. In addition, the General Medical Services (GMS) card, which is available to all persons aged ≥ 70 years and those under 70 years who meet a certain income threshold, facilitates the use of the majority of health services free of charge, including GP practice visits as well as inpatient, emergency and outpatient services in public hospitals. In this group, medication charges incur a small co-payment (€2.50 per item) to a maximum of €25 per family per month [9]. Those not eligible to receive a medical card must pay for primary care following each visit. In addition, the two jurisdictions differ in their stage of adopting a national ageing strategy and implementation of integrated health and social care [6, 7].

### Study population and recruitment

Overall, a total of 64 patients will be recruited (32 per randomised group), with eight patients recruited from each participating general practice. The inclusion criteria are: (1) aged ≥ 70 years; (2) being in receipt of a valid GMS card in the RoI, or registered for NHS primary care services in Northern Ireland; (3) have two or more chronic medical conditions (multimorbidity); (4) prescribed four or more regular medications; and (5) able to complete an English language postal questionnaire. The exclusion criteria are: (1) receiving specialist palliative care; (2) a record of assessed cognitive impairment at the level that would impact their ability to complete screening postal questionnaire, outcome measures and participate in a patient care conference(s) (defined as Mini Mental State Examination (MMSE) ≤ 20); (3) experiencing a psychotic episode at the time of recruitment; or, (4) hospitalised long-term, in a nursing home, homeless or in sheltered accommodation.

#### Patient screening

The GP practice manager will conduct a search employing the study eligibility criteria on the practice’s electronic health record system to identify potential study participants. Eligible patients will be sent a letter from their GP informing them about the project and inviting them to complete the PRISMA 7 questionnaire. This is a seven-item screening questionnaire that includes items related to age, gender, mobility, need for assistance in activities of daily living and the availability of informal support [10]. It identifies frailty, is suitable for postal completion and is considered a best-practice tool recommended by NHS England to case find at-risk patients for frailty in general practice. Individuals who obtain a score of > 3 are identified as being at risk of functional decline [11, 12]. If patients do not respond to an initial invitation letter, one follow-up reminder will be mailed 7 to 10 days later.

#### Patient enrolment

The study methodology is designed to identify individuals who will screen as at risk of functional decline. Initially, patients who screen as ‘at risk’ would receive a letter from their GP inviting them to participate in the study. Patients who reply that they agree to be contacted will be telephoned by a research assistant, who would seek their consent by telephone. Allocation to the intervention versus the usual care group will be communicated to the study participant by a member of the research team after consent has been obtained and the baseline-standardised interview completed. If more than one eligible participants is identified in any one household, all will be eligible for enrolment into the study and, if enrolled, would receive the same study allocation. Following consent, study nurses will commence arrangements to visit the study participants in the intervention group. Changes to enrolment procedures in the RoI were made in response to ethical review in that jurisdiction. These changes related to patient screening and require patients who meet the study inclusion criteria to be assessed for risk of functional decline through a three-step process: (1) GP staff generate a pseudonymised list of potential participants via the GP electronic health record system search, identifying candidates who have two or more chronic medical conditions; four or more regular prescribed medications and, the availability of an informal caregiver if recorded; (2) identified candidates will be reviewed by their own GP to confirm suitability for inclusion in the study; and, (3) eligible patients will then be sent a letter from their GP providing information about the study and inviting them to complete the PRISMA 7 questionnaire that will also include a ‘consent to be contacted’ statement. Patients who do not respond to the initial GP’s letter may be contacted using the same methodology on one additional occasion. If there is no response at this stage, they will not be contacted again. Patients return the completed pseudonymised PRISMA 7 to the research team which will then score it. The research team will then send the GP practice staff the unique code assigned to the completed PRISMA 7 questionnaire. These staff members will link the patient’s name, telephone number and address to the assigned code. Patients who do not screen as ‘at risk’ on the basis of their answers on the PRISMA 7 questionnaire will receive a letter from the GP explaining that they do not meet the criteria to participate in the study and thanking them for their time. The GP practice manager will provide the research team with the name and telephone number of patients who do screen as at risk and have consented to be contacted by them. A member of the research team will then telephone the patient to discuss the study and answer any questions that they have. If a patient is interested in participating in the study, the researcher will arrange to meet with them to complete the written informed consent process and administer baseline questionnaires.

#### Sample

A total of 64 patients (32 per randomised group) will be enrolled into the study. This will comprise eight patients per GP practice, and random selection will be used if more than eight patients are available in a practice. This size of sample is recommended to allow the standard deviation (SD) of the continuous outcome EuroQol 5-dimension, 5-level health survey (EQ-5D-5 L) [13] to be determined at a sufficient level of accuracy while minimising the number of patients required in the pilot [14, 15].

#### Nurse training

The framework for this intervention comprises personalised care and support planning. To ensure a personalised care approach in the ACP intervention, registered nurses from both jurisdictions complete a training programme that was designed to orientate the nurses to the intervention and study procedures. This training lasts for 3 days and is facilitated by a clinician who is acknowledged as an expert in the field. It covered a range of topics including: an overview of the study; principles and practice of personalised care; shared-decision-making; conducting a holistic assessment using the Easy-Care Assessment instrument [16]; and, completing a medication review facilitated by a clinical pharmacist.

### The intervention group

As a first step in the intervention, the study nurse will contact the patient by telephone to schedule an initial home visit. During that visit, the study nurse will complete an ACP assessment with the aid of a medical summary provided by the GP practice. This will include details of the patient’s health conditions and currently prescribed medications. The ACP assessment will be conducted using the EASY-Care assessment tool to ensure that a personalised holistic approach is used and this assessment will be supplemented with a medication review. A personalised care approach will encourage discussion with the patient and their family carer about present and future care and patient goals. The patient will be asked to prioritise any concerns that they have in order to guide the research nurse in the development of a person-centred care plan.

Following the initial home visit, the study nurse will draft a structured summary report of the assessment that will include patient goals, preferences for care, identified problems and an action list. The study nurse will forward the medications review to the study pharmacist who will conduct a desk-based evaluation based on established guidelines [17] to determine medication management considerations to be brought forward to the patient’s GP. Subsequent to the pharmacist review, the study nurse will finalise the summary report and will meet with the patient’s GP who will be informed through a structured format of the patients identified goals and wishes, the results of the patient assessment, the problem list and recommended actions. The GP will review, provide feedback and confirm their agreement with the suggested plan of care.

Following the GP consultation, the study nurse will, depending on the complexity of the identified care needs or functional needs of the patient, either meet with the patient and family carer again or contact them by telephone. During this meeting or telephone call the study nurse will confirm and discuss the patient’s identified priorities and then initiate a discussion of identified options for support. While the number and frequency of visits will vary depending on the complexity of a patient’s needs, it is expected that participants in the intervention group will receive up to 5 h of nursing contact over 10 weeks. This will include the initial home visit to complete the holistic assessment, meetings with the GP, contacts with other health and social care providers and any follow-up home visits and telephone contacts.

There will be no special criteria for discontinuing or modifying the allocated interventions.

#### Usual care group

Patients in the usual care group will not receive the ACP intervention but will receive usual care from their GP. The PRISMA 7 score and the explanation of this score will not be shared with the GP in this group. Usual care comprises the following: patients seeking appointments with their GPs to discuss presenting complaints as these arise, if any. Given the range and complexity of potential presenting issues, an exhaustive account is not provided here. This comparator is, therefore, usually reactive, rather than anticipatory. However, some care episodes will be initiated by the GP, e.g. a reminder for the influenza vaccine, chronic care delivery for certain chronic conditions such as diabetes, reminders regarding need for monitoring bloods for certain medical conditions/medications. GPs fulfil a gatekeeper role to secondary care, and access to care is free for older patients who have a medical card in the RoI. Similar to participants in the intervention group, usual care participants will complete study questionnaires with the help of a research assistant at baseline, 10 weeks and 6 months following enrolment.

There is no anticipated harm or compensation for trial participation.

### Data collection

The RE-AIM conceptual framework will guide considerations for this evaluation of the ACP intervention [18–20]. Four of the five factors included in the RE-AIM framework apply to the parameters of this feasibility cRCT study: (1) *R*each – describes the number, proportion and representativeness of general practices and patients who participate in the initiative; (2) *E*ffectiveness – describes the impact of the intervention on outcomes; (3) *A*doption – examines the willingness of general practices exposed to the intervention who are willing to initiate the intervention; and (4) *I*mplementation – explores the fidelity of the intervention protocol and the consistency of its implementation across primary care practices and jurisdictions. In most cases, the *M*aintenance component of the RE-AIM framework refers to on-going implementation into routine care and is, therefore, beyond the scope of this feasibility study.

### Patient standardised interview (RE-AIM Effectiveness factor)

On obtaining consent from the patient to participate in the study, a baseline patient standardised interview will be conducted by a trained project research assistant in the patient’s own home. In order to assess the impact of the intervention, all patients will participate in the individual standardised survey interviews at baseline, and at their 10-week and 6-month follow-ups. Home interviews last for approximately 1 h.

### Baseline measures

Information on variables expected to predict responsiveness to the intervention (demographic factors, social support, medical conditions, prescribed medications, and cognitive impairment) will be obtained during the initial (baseline) home interview with the research assistant:

1. *Demographic data*. Demographic data will include age, gender, education, living arrangements, income and economic resources
2. The Medical Outcomes Study Social Support Survey (MOS) [21], which is a 20-item instrument designed for use with chronically ill patients, will be used to assess four categories of social support: tangible support, affectionate support, positive social interaction, and informational support. Respondents use a Likert-type scale to rate each item ranging from ‘None of the Time’ to ‘All of the Time’. Psychometric properties of the measure are sound. It has demonstrated good reliability, internal consistency and construct validity

### Outcome measures

To evaluate the ACP intervention, a mixed-methods approach consisting of both quantitative measurement and qualitative interviews will be applied. All quantitative outcome measures will be assessed at baseline, 10 weeks and 6 months. All selected measures have been used with this population in previous research as both outcome and observational measures [22–24].

### Primary outcome measures (RE-AIM – Effectiveness factor)

1. The *EQ-5D-5 L* [13] is a widely used self-reported generic measure of health-related quality of life that has been validated in different patient populations. The five-level version contains the same dimensions as the earlier three-level version but has been designed to provide greater reliability and sensitivity
2. *Center for Epidemiological Studies Depression Scale (CES-D)* [25] is a 20-item scale that has been used extensively with older adults. Respondents use a 4-point scale to rate how they have felt in the past week in relation to symptoms associated with depression

### Secondary outcome measures (RE-AIM – Effectiveness factor)

1. *The Patient Assessment of Chronic Illness Care (PACIC) Scale* [26] is a 20-item scale was designed to assess, from the patient’s perspective, the receipt of patient-centered care and self-management behaviours
2. *Health economic evaluation*. The health economic analysis in this feasibility study will consist of a trial-based economic evaluation and will incorporate both cost-effectiveness analysis and cost-utility analysis to compare the ACP Intervention versus usual care in general practice. The basic tasks of the evaluation are to identify, measure, value and compare the costs and outcomes of the alternatives being considered. This feasibility study will report on the process to identify the appropriate resource use items to be included and on the appropriate methods for collecting this data. Further, the study will consider the feasibility of conducting the incremental analyses and report on the potential cost-effectiveness data generated. Evidence collected on resource use and outcome measures alongside the study will provide the basis for the analysis over the follow-up period. With respect to costing, a health service perspective will be adopted. Resource use associated with delivery of the ACP intervention will be measured and costed. Other resource use to be captured will include usage of medications, primary care, community care and hospital care services. For the cost-effectiveness analysis, the ACP intervention and usual care will be compared on the basis of the effectiveness data for the primary outcomes of interest. For the cost-utility analysis, effectiveness will be evaluated on the basis of Quality Adjusted Life-Years (QALYs) which will be estimated using data obtained with the EuroQol EQ-5D-5 L survey instrument [13]. The health economic analysis will employ the standard approach for the comparison of alternative treatment strategies in terms of costs and health outcomes. An incremental analysis will be undertaken to provide information on the marginal costs and effects of the ACP intervention relative to usual care through the calculation of incremental cost-effectiveness ratios and incremental net benefits. Uncertainty in the incremental analyses will be explored using cost-effectiveness acceptability curves
3. The *Katz Index of Independence in Activities of Daily living* [27] is a well-established measure of performance. It describes what the person actually does, rather than what they are capable of doing. It assesses six activities: ability to bathe, dress, toilet, transfer, feed yourself and maintain bowel and bladder continence. A three-category scoring model is used for each activity
4. The *Generalised Anxiety Disorder 7 (GAD-7)* [28] is an established measure which asks patients how often in the last 2 weeks they had been bothered by seven anxiety-related symptoms. It is a valid and efficient tool for screening for generalised anxiety and assessing its severity
5. *Medication management* [29] will be assessed following the medication review conducted by the study nurse in collaboration with the study pharmacist. We will report the proportion of patients who receive recommendations from the pharmacist following their medications review. We will assess the mean number of suggested changes to prescribed medications

Adverse events are not anticipated. If any occur, they will be reported to the relevant regulatory bodies as required.

1. Tracking intervention patterns and intensity (RE-AIM Implementation) During the trial implementation, the Project Management Committee will meet weekly, while a Project Oversight Committee will meet quarterly to review processes and conduct.
  Records will be maintained describing the patterns and intensity of care provided to each participant in the ACP intervention group. Nurses will maintain logs to record the amount of time spent with each participant including home visits, telephone calls and consultations with family physicians and other professionals
2. Process evaluation (RE-AIM – Adoption)
  In addition to the collection of quantitative data, a qualitative approach will also be pursued. User perceptions on the appropriateness, benefits and convenience of the ACP intervention will be recorded through interviews. Patient acceptability of the ACP intervention will be assessed using several additional questions embedded in the semi-structured interview schedule conducted at the 10-week follow-up for the 32 participants in the ACP intervention group (*n* = 32). These questions will assess perceptions about the intervention in terms of: (a) the overall intervention; (b) its component parts (the patient meetings, assessment, patient education on advance care planning); (c) implementation (was the home environment suitable for meetings); (d) whether the contents reviewed in the meetings were useful; and (e) suggestions for refining the intervention in future. The five study nurses will be interviewed on aspects of the intervention to review: (a) what experience a registered nurse should have to fulfil the position; (b) training requirements; (c) how to build relationships with participants; (d) if the home environment was suitable for the meetings; (e) if the ACP model fitted into the running of a GP practice; (f) their best and worst experience related to ACP meetings; and (g) any recommendations that they would make to improve the ACP intervention. GP practice staff (GPs and associated healthcare providers, e.g. practice nurse and practice manager) (estimate *n* = 12) will be interviewed to examine their perceptions on the appropriateness, benefits and convenience of the ACP intervention. In addition, community health professionals (estimate *n* = 12) will be interviewed to identify any facilitators and barriers at the regional healthcare system level that may influence the manner in which the ACP intervention is implemented.
3. Metrics to be collected (RE-AIM – Reach)
  Records will be maintained that will collect indicators on: (a) number of eligible participants identified on the GP patient list; (b) number of potential participants found to be ineligible and reasons why; and (c) number of eligible participants who do not consent to participate and reasons for non-participation.

### Criteria for progression to a full trial

A protocol for a definitive trial will be developed if this study’s findings demonstrate that the ACP intervention is acceptable to most (> 70%) patients, their carers and health professionals; if the ACP is perceived by GP practice staff to be readily implemented; if > 50% of eligible patients are recruited and > 65% of recruited patients are retained; if there is a detected difference in the primary and secondary expected outcomes between the intervention and usual care groups; and the features of the economic evaluation are found to be feasible [30].

### Data analysis

#### Quantitative analysis

Data will be expressed as a mean, SD or median and for continuous variables and count (percent) for categorical variables. The analysis of indicators will be based on descriptive statistics reported as estimates with confidence intervals. Outcome analyses will be conducted to compare the intervention and control groups, recognising that this analysis will be underpowered for a robust statistical analysis. Means and SD will be reported for each combination of baseline and final data. Frequency and percentage will be reported in the same manner for categorical variables. The SD of the difference in primary outcome (EuroQol EQ-5D-5 L at 6 months) will be determined and the intra-cluster correlation of this difference will be estimated to inform the sample size required for the definitive trial. Recruitment and retention rates at 6 months will also be used to inform the sample size calculation. We will also consider acceptability of the mode and timing of the administration of impact measures. Incremental cost-effectiveness and cost-utility analyses will be conducted for the purposes of the economic evaluation. All quantitative analysis will be conducted in a manner consistent with guidelines for the analysis of data from cRCTs. The data analyst will be blinded for all primary and secondary quantitative outcome analysis, with the exception of the health economics analysis. All participants will be analysed according to the intention-to-treat (ITT) principle. The primary analysis will be a complete case analysis and a sensitivity analysis will be conducted using multiple imputation to impute missing values. A Data Monitoring Committee was not considered as this was a low-risk intervention. There are no stopping guidelines as there are no anticipated problems that are detrimental to the participants.

#### Qualitative analysis

The software package NVivo 10.0 [31] will be used to help organise and analyse the qualitative data. We will analyse interview data following the template analysis style outlined by Miles and Huberman [32] and will develop an open-ended and modifiable codebook. We will use this tool to generate themes, patterns and interrelationships in an interpretive fashion, drawing on the expertise of our research team and our Personal and Public Involvement (PPI) advisors. *No blinding for qualitative analysis is possible*.

### Ethical considerations

Ethical approval was obtained in the RoI from the Research Ethics Committee, the Irish College of General Practitioners (reference: ICGP2018.4.10, date of approval: 28 January 2019; and, in Northern Ireland from the Office for Research Ethics, Northern Ireland (ORECNI) I (reference: 19/NI/0001. IRAS Project ID: 247572), date of approval; 7 February 2019. All participating GP practices completed a letter of support confirming that they had appropriate insurance to cover their staff members’ participation in the study. In addition, general practices also completed a data-sharing agreement to guide the processing and provision of personal data under the terms of the General Data Protection Regulation (GDPR) and the Data Protection Act (DPA) 2018.

## Discussion

This study will inform the feasibility for a definitive trial to evaluate the implementation and outcomes of an ACP intervention in primary care to assist older adults aged > 70 years and assessed as being at risk of functional decline. Recent systematic reviews have failed to identify any trials of anticipatory and integrated care on the island of Ireland [33, 34], so this study will be the first to determine the feasibility of evaluating an ACP intervention on the island of Ireland. This study will span two jurisdictions with different health systems but similar health challenges, providing novel findings. Important issues, such as different interpretations of legislation such as General Data Protection Rules, can be explored during the implementation of the study, and will provide vital learning for other cross-border trials on the island of Ireland.

From a methodological perspective, due to the different nature of the healthcare systems in the RoI and Northern Ireland and the potential for contamination associated with individual patient randomisation, we opted for a cluster-randomised trial in both jurisdictions. The cross-border cooperation exemplified in this trial should provide an evidence-based assessment on the potential impact of the ACP intervention on patient quality of life, healthcare utilisation, costs and appropriate prescribing in an all-Ireland context. The cross-border approach should also identify optimal system delivery supports for effective implementation and impact of the ACP intervention. Overall, our findings will inform the feasibility of developing a large, cross-border trial on ACP for older adults on the island of Ireland.

## Trial status

The study started in April 2019. Protocol version number 2, approved in January and February 2019 (see above). At the time of writing (September 2019), recruitment and baseline data collection are in progress, with the final participants expected to be recruited in October 2019, while we expect the results of the cRCT to be published in 2020.

## Supplementary information

**Additional file 1.** World Health Organisation (WHO) Registry Checklist.

**Additional file 2.** Standard Protocol Items: Recommendations for Interventional Trials (SPIRIT) Checklist.

## Data Availability

Not applicable for this protocol. On study completion the study materials and ultimately the aggregated data and statistical code will be available from the lead author on reasonable request.

## Abbreviations

ACP: Anticipatory Care Planning
CES-D: Centre for Epidemiological Studies Depression scale
cRCT: Cluster randomised controlled trial
DPA: Data Protection Act
GAD-7: Generalised Anxiety Disorder–7 questionnaire
GDPR: General Data Protection Regulation
GMS: General Medical Services
GP: General practitioner
ICGP: Irish College of General Practitioners
MMSE: Mini Mental State Examination
MOS: Medical Outcomes Social Support Survey
NHS: National Health Service
NI: Northern Ireland
ORECNI: Office for Research Ethics, Northern Ireland
PACIC: Patient Assessment of Chronic Illness Care scale
QALY: Quality Adjusted Life-Years
RoI: Republic of Ireland
SD: Standard deviation
