# Supplementary material for: Anticipatory care planning intervention for older adults at risk of functional decline: study protocol for a primary care cluster feasibility randomised trial
Source: Trials. 2020 Feb 11;21:168. doi: 10.1186/s13063-020-4100-2 (PMC7014651; doi:10.1186/s13063-020-4100-2)
Supplement: Supplementary file 1 — Additional file 1. World Health Organisation (WHO) Registry Checklist. [file 13063_2020_4100_MOESM1_ESM.docx]

Additional File 1: Items from the World Health Organization Trial Registration Data Set

| Data category | Information |
| --- | --- |
| Primary registry and trial identifying number | ClinicalTrials.gov NCT03902743 |
| Date of registration in primary registry | 2 April 2019 |
| Secondary identifying numbers | Not applicable |
| Source(s) of monetary or material support | Cross-border Healthcare Intervention Trials in Ireland Network (CHITIN) a programme funded by the European Regional Development Fund (award number: CHI/5426/2018) |
| Primary sponsor | Cross-border Healthcare Intervention Trials in Ireland Network (CHITIN) a programme funded by the European Regional Development Fund (award number: CHI/5426/2018) |
| Secondary sponsor(s) | Cross-border Healthcare Intervention Trials in Ireland Network (CHITIN) a programme funded by the European Regional Development Fund (award number: CHI/5426/2018) |
| Contact for public queries | Kevin Brazil, [k.brazil@qub.ac.uk](mailto:k.brazil%40qub.ac.uk)  David Scott, [david.scott@qub.ac.uk](mailto:david.scott%40qub.ac.uk) |
| Contact for scientific queries | Kevin Brazil, [k.brazil@qub.ac.uk](mailto:k.brazil%40qub.ac.uk)  David Scott, [david.scott@qub.ac.uk](mailto:david.scott%40qub.ac.uk) |
| Public title | Anticipatory Care Planning Intervention for Older Adults at Risk of Functional Decline: A Primary Care Feasibility Study |
| Scientific title | Anticipatory Care Planning Intervention for Older Adults at Risk of Functional Decline: A Primary Care Feasibility Study |
| Countries of recruitment | Northern Ireland, Republic of Ireland |
| Health condition(s) or problem(s) studied | 1) aged ≥70 years; 2) in receipt of a valid general medical services (GMS) card in the Republic of Ireland, or for Northern Ireland registered for NHS primary care services; 3) ability to complete a postal questionnaire. |
| Intervention(s) | The Anticipatory Care Plan will be delivered by a trained nurse. At the initial visit, the nurse will conduct a hollistic assessment using the EasyCare Assessment Tool. Following this visit, the nurse will draft a strucutred summary report of the home visit to include patient goals, preferences for care, identified problems and action list. The nurse will then consult with a pharmacist to identify any potential inappropriate prescribing. The nurse will then meet with the participants GP and review and provide feedback. The nurse will then re visit participants and discuss the report and agree a support plan and actions for follow up. |
|  | Usual care |
| Key inclusion and exclusion criteria | Ages eligible for study: ≥70 years Sexes eligible for study: both Accepts healthy volunteers: no |
|  | Inclusion criteria: 1) aged ≥70 years; 2) in receipt of a valid general medical services (GMS) card in the Republic of Ireland, or for Northern Ireland registered for NHS primary care services; 3) ability to complete a postal questionnaire |
|  | 1) receiving specialist palliative care; 2) record of assessed cognitive impairment at the level that would impact their ability to complete screening postal questionnaire, outcome measures and participate in a patient care conference(s) (defined as Mini Mental State Examination (MMSE) ≤20); 3) experiencing a psychotic episode at the time of recruitment; 4) hospitalised long-term, in a nursing home, homeless or in sheltered accommodation. |
| Study type | Interventional |
|  | Allocation: randomized intervention model. Parallel assignment masking: none |
|  | Primary purpose: Health Services Research |
|  | Feasibility cluster trial |
| Date of first enrolment | April 2019 |
| Target sample size | 64 |
| Recruitment status | Recruiting |
| Primary outcome(s) | EQ-5D-5L [ Time Frame: 10 minutes ]  Is a widely used self-reported generic measure of health reported quality of life that has been validated in different patient populations. The five-level version contains the same dimensions as the earlier three-level version but has been designed to provide greater reliability and sensitivity.  Center for Epidemiological Studies Depression Scale (CES-D) [ Time Frame: 10 minutes ]  This 20- item scale has been used extensively with older adults. Respondents use a four choice scale to rate how they have felt in the past week expressing depressed mood. |
| Key secondary outcomes | - Patient Assessment of Chronic Illness Care (PACIC) Scale [ Time Frame: 15 minutes ]   This 20 item scale was designed to assess from the patient perspective the receipt of patient-centered care and self-management behaviors.   - Health Economic Evaluation [ Time Frame: 15 minutes ]   The health economic analysis will consist of trial-based economic evaluation and will incorporate both cost effectiveness analysis and cost utility analysis to compare the alternative treatment strategies: (1) the Anticipatory Care Planning (ACP) Intervention; and (2) usual care in general practice. |
